# Triangulating multimodal data: Data of interaction logs, learning achievement, and motivation of L2 learners in a computer-assisted language learning environment

**DOI:** 10.1016/j.dib.2025.112426

**Published:** 2025-12-24

**Authors:** Mihwa Lee, Björn Rudzewitz, Yushan Ye, Lanhua Huang, Yuan Chu, Xiaoer Zhou, Xiaobin Chen

**Affiliations:** a Hector Research Institute of Education Sciences and Psychology, University of Tübingen, Walter-Simon-Straße 12, Tübingen, 72072, Germany; b LEAD Graduate School & Research Network, University of Tübingen, Europastraße 6, Tübingen, 72072, Germany; c School of Foreign Languages, Guangzhou City University of Technology, No.1 Xuefu Road, Huadu District, Guangzhou 510800, China

**Keywords:** Digital trace data, Learning analytics, Computer-assisted language learning, Motivation

## Abstract

This dataset comprises detailed interaction log data from 201 learners engaged in second language (L2) English reading assignments with an intelligent computer-assisted language learning (ICALL) system over a six-week period. After preprocessing, a total of 116,168 clickstream data points were generated, capturing learners’ behaviours, such as navigation patterns and task engagement within the system. In addition to the interaction logs, the dataset includes learners’ L2 reading proficiency test scores collected before and after the learning period and self-reported measures of motivation toward the subject. These multimodal data provide valuable insights into how learners engage with online learning materials, how person-level factors such as motivation interact with digital reading behaviours, and how digital traces can be used to predict learning achievement.

**Table:** Specifications Table.

Subject	Social Sciences
Specific subject area	Interaction logs, test scores, and survey data collected in an ICALL system
Type of data	Table Text (test and survey items) Raw Filtered Processed
Data collection	The dataset was collected online through an ICALL system *ARES* between November 2024 and January 2025. It consists of students’ detailed interaction logs, scores frompre- and post-tests, and responses to a motivation questionnaire from 201 undergraduate students. The system was implemented as part of the regular teaching and learning routine for a six-week period. During this time, teachers assigned at least two L2 reading assignments per class each week. The assignments were typically accompanied by a few reading comprehension questions in the form of multiple choice questions or/and short open-ended questions about the text. Submissions were evaluated by the corresponding teacher using the system. The pre-test for L2 English reading proficiency and the motivation questionnaire were administered before the learning period on the system, and the post-test after the learning period.
Data source location	University of Tübingen, Tübingen, Germany
Data accessibility	Repository name: OSF Data identification number: 10.17605/OSF.IO/8Y3ZP Direct URL to data: https://osf.io/8y3zp/?view_only=118d77f5f62940df9561cb4e7a397522
Related research article	Not applicable

## 1 Value of the Data

• The dataset provides valuable insights into how students engage with digital L2 reading assignments and their navigational patterns within an ICALL system through interaction logs. This information can help educators and researchers understand students’ engagement and learning strategies enacted during the learning process, as well as develop instruction strategies based on individual engagement patterns and learning style preferences.
• In addition to interaction logs, the data includes L2 English reading proficiency scores and self-reported measures of motivation. This set of data allows researchers to triangulate multimodal data to examine how behavioral factors and person-level factors (e.g., motivation) jointly influence learning outcomes.
• Educational data mining (EDM) and Learning Analytics (LA) researchers can benefit from this dataset by developing predictive models and profiles of learner performance, engagement, and motivation in digital language learning environments.
• The dataset can also benefit educators and learning system designers by supporting the development of adaptive learning interventions and personalized feedback mechanisms based on individual learning styles and the influence of person-level factors on learning engagement patterns.

## 2 Background

The rise of synchronous and asynchronous online learning offers new opportunities for both instructors and students, including access to learning resources, interactive multimedia, and collaborative learning experiences beyond time and location [1,2]. A key advantage of online learning is that students’ behaviour can be tracked through digital trace data, unobtrusively capturing large-scale students’ interactions [[3], [4], [14]), without the drawbacks of subjective measures (e.g., surveys) or labor-intensive approaches (e.g., interviews). Trace data therefore serve as a valuable proxy for the time and effort students invest in learning activities [[5], [13]].

Such trace data can be linked with complementary measures, such as self-reports and performance assessments, to gain a more comprehensive understanding of learner engagement in learning and related constructs. For instance, how do psychological antecedents (e.g., motivation, learning beliefs) relate to specific learning behaviours? Which behaviours predict outcomes? How do behaviours mediate the effect of psychological factors on learning? Triangulating multiple data sources allows for richer insights, helping align behavioural traces with other constructs such as motivation and performance to inform instructional design and adaptive support [6].

## 3 Data Description

The data presented in this article were collected from 201 foreign language (L2) learners engaged in digital L2 English reading assignments in an intelligent computer-assisted language learning (ICALL) environment. The participants were drawn from seven English as a foreign language (EFL) classes in a four-year bachelor program at a Chinese university and all students majored in English. The dataset consists of seven files containing learners’ interaction logs, responses and scores from pre- and post-tests of L2 English reading skills, and self-reported motivation toward the subject. Table 1 summarizes the description of the data contained in each file. In the following sections, we outline the details of each data.

**Table 1:** Summary of data contained in each file.

File name	Description
*xapi_statement_student.xlsx*	Logs of students’ interaction with the system
*pre_test_response.xlsx*	Individual responses from the pre-test on L2 English reading proficiency
*pre_test_score.xlsx*	Scores from the pre-test on L2 English reading proficiency
*post_test_response.xlsx*	Individual responses from the post-test on L2 English reading proficiency
*post_test_score.xlsx*	Scores from the post-test on L2 English reading proficiency
*pre_motivation_response.xlsx*	Individual responses from the motivation questionnaire
*pre_motivation_score.xlsx*	Scores of subscales from the motivation questionnaire

### 3.1 Interaction logs

**Fig. 1:**
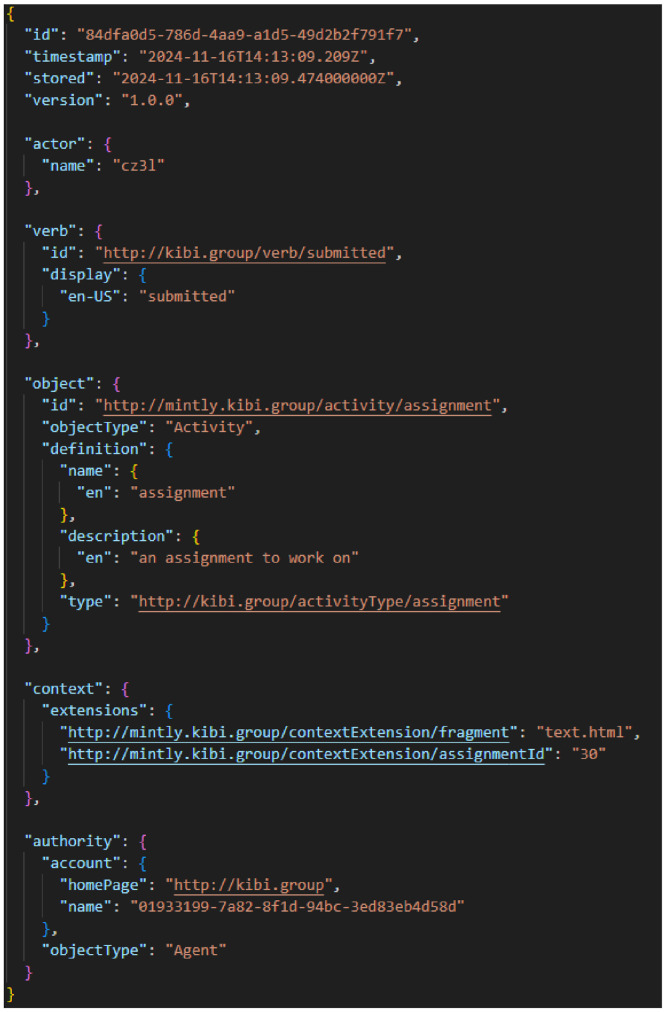
An example of a xAPI statement representing a learning activity.

The file “xapi_statement_student. xlsx” contains logs of students’ interactions with the system. After preprocessing, a total of 116,168 data points were recorded in the interaction logs. All user activities, such as button clicks, vocabulary lookups, reading comprehension question attempts, assignment submissions, and viewing of specific feedback messages, were logged using the Experience API (xAPI). xAPI^1^ is a specification designed to enable interoperability of learning experience data from diverse educational sources or applications. It provides a standard data model for recording students’ learning experiences and an API for sharing this data among systems for analysis. As shown in Fig. 1, each event in xAPI is captured as a statement in a standardized format: [actor] [verb] [object] [context]. In the example in Fig. 1, the xAPI states that “a user cz3l (actor) submitted (verb) an assignment (object) with an assignment ID 30 (context)”. Following this specification, the file contains five columns including actor (a four-character alphanumeric pseudonymized user ID, e.g., Q94J), verb (the action the actor enacts, e.g., logging-in, entering, pressing, opening), object (the object on the action is executed, e.g., an assignment page, a popup displayed on the screen, vocabulary in a reading text), context (additional information of the context where the action was taken, e.g., assignment ID, vocabulary ID), and timestamp (the time that the user’s action was sent to the server, e.g., 2024–11–10 12:29:13). The file was preprocessed to make the data analysable by formatting the raw xAPI statements (see the section EXPERIMENTAL DESIGN, MATERIALS AND METHODS for the data preprocessing steps). Table 2 lists the vocabulary used in the log statements following the xAPI specification.

**Table 2:** List of the vocabulary used in interaction logs using the xAPI specification.

Vocabulary	Description
Verb	
logged-in	A user logs in to the system
entered	A user enters a fragment (e.g., page)
exited	A user exits a fragment (e.g., page, popup)
enrolled	A user enrolls themselves in a class a teacher created
unenrolled	A user unenrolls themselves from a class
pressed	A user presses a button
opened	A user opens a fragment of a page (e.g., page, popup)
focused	A user focuses on a text input field (e.g., open-ended reading comprehension question)
submitted	A user submits an assignment
Object	
page	Page (e.g., home page, assignment overview page, enrollment page, assignment text page)
assignment	Assignment assigned in the enrolled class
btn	Button (e.g., submission button, exit button, feedback view button)
filter	Filtering function (e.g., assignment status, class)
klass	Class existing in the system
token	Word in a reading text (e.g., “playwright” in a sentence “He was a renowned English playwright, poet, and actor.”)
construct	Grammatical construct associated with a word in a reading text
word-explanation	Vocabulary explanation associated with a word in a reading text
dialog	Popup (e.g., submission confirmation popup, warning popup for empty answers)
txtView	Text input field (e.g., open-ended reading comprehension question)
response	Response to the assignment reflection (e.g., difficulty and interestingness of a reading text)
evaluation	Evaluation of a submitted answer in an assignment (e.g., feedback, score)
Context	
fragment	Page name (e.g., assignment page, home page)
assignmntId	ID of an assignment
textId	ID of a reading text
btnDiscription	Description of a button (e.g., button to finish an assignment)
txtViewDescription	Description of a text input field (e.g., question ID associated with a reading comprehension question)
klassId	ID of a class
dialogDescription	Description of a popup (e.g., popup for alerting an empty answer)
accessCode	Access code for enrolling in a class (e.g., DH89)
submissionId	ID of a submission
filterName	Type of a filter (e.g., assignment status, class)
filterValue	Value of the filter (e.g., assignment: not submitted yet, submitted, evaluated; class: Class A5, Class B1)
token	Word in a reading text (e.g., “playwright” in a sentence “He was a renowned English playwright, poet, and actor.”)
tokenId	ID of a word in a reading text
annotation	List of grammar construct IDs related to the word in a reading text
constructId	ID of a grammar construct
constructLevel	Level of a grammar construct in CEFR (e.g., A1, B2, C2)
constructType	Type of a grammar construct (e.g., nouns after prepositions)
wordContext	Context of a word (e.g., He was a renowned English playwright, poet, and actor.)
questionId	ID of a question
questionsContent	Content of a question (e.g., what is the main message the author wanted to convey?)
mcOptionId	ID of an option of a multiple choice question
mcOptionContent	Content of an option of a multiple choice question (e.g., b)
evaluationId	ID of an evaluation to the submitted answer
evaluationContent	Content of an evaluation to the submitted answer (e.g., “Good job! You got it right!”)
evaluationRead	Whether an evaluation to the submitted answer was already read or not (“true” = evaluation was read, “false” = evaluation was not read yet)
answerId	ID of an answer submitted by a student
answerCorrect	Correctness of an answer submitted by a student (“true” = answer is correct, “false” = answer is incorrect)
answerContent	Content of an answer submitted by a student
ownScore	Student’s own score on an assignment (e.g., 75 %)
classScore	Average class score of an assignment (e.g., 80 %)
responseType	Type of a response (e.g., test, questionnaire)
ratingName	Type of a question of an assignment reflection (e.g., difficulty and interestingness of a reading text)
ratingValue	Value of an assignment reflection (e.g., 3 out of 5 in a 5-Likert scale)

### 3.2 Pre- and post-tests

There are four files containing data on pre- and post-tests: *pre_test_response.xlsx, post_test_reponse.xlsx, pre_test_score.xlsx*, and *post_test_score.xlsx*. The first two files contain each learner’s individual responses to the questions of the tests while the last two files contain only each learner’s total test scores. The response files include five columns: *user_id* (a four-character alphanumeric pseudonymized user ID), *prompt* (test item, e.g., PreTestQ1), *response* (the answer the user selected, e.g., c), *correct* (correctness of the selected answer; “t” indicates a correct answer was selected and “f” means the selection was incorrect), and *timestamp*. The score files include two columns: *user_id* and *score* (total score of the test).

### 3.3 Motivation questionnaire

Two files contain data from the motivation questionnaire: *pre_motivation_response.xlsx, pre_motivation_score.xlsx*. Similar to the test files, the response file contains each learner’s individual responses of the questionnaire with four columns: *user_id, prompt* (questionnaire item, e.g., PreMotiQ1), *response* (the scale the user selected on a 7-likert scale, e.g., 4), and *timestamp*. The score file contains each learner’s total scores of each subscale of the questionnaire with seven columns: *user_id, intrinsic_goal_orientation, extrinsic_goal_orientation, task_value, control_learning_beliefs, self_efficacy*, and *test_anxiety*.

## 4 Experimental Design, Materials and Methods

### 4.1 Procedure

**Fig. 2:**
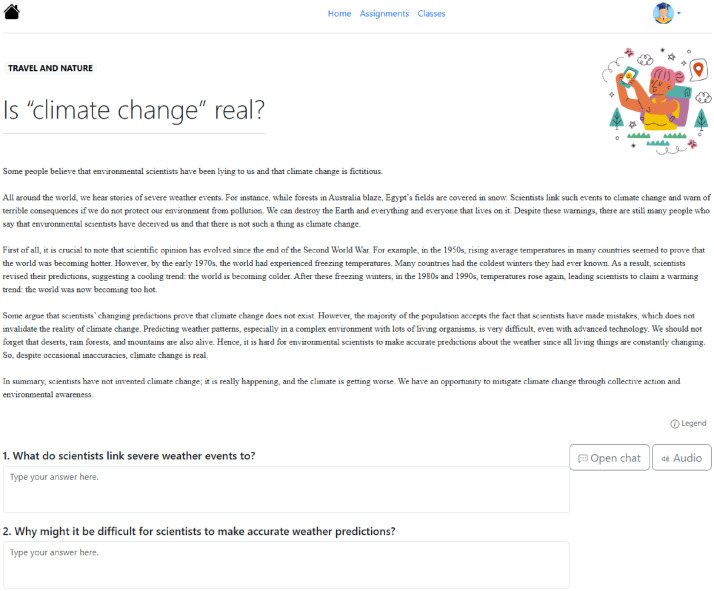
Reading interface of the *ARES* system.

The data were collected between November 2024 and January 2025 involving seven EFL classes in a four-year bachelor program at a Chinese university. All the students majored in English. Students provided informed consent for participation in the study and the scientific use of their data. After removing data for which no consent was given by the students or users that had no activity recorded in the system, the final sample consisted of 201 students in the first, second, and third years of the program. The study was conducted entirely online using an ICALL system called *Annotated Reading Enhancement System* (*ARES*, see the next section and Lee et al. [7] for details). At the beginning of the study, teachers and students were introduced to how to use the system. The system was implemented as part of the regular teaching and learning routine for a period of six weeks. In order to maximize the ecological validity, we did not restrict how students or teachers used the system. The only requirement was that teachers assign at least two reading assignments per week per class. The assignments were typically accompanied by a few reading comprehension questions in the form of multiple choice questions or/and short open-ended questions about the text (see Fig. 2). Students had the flexibility to work on and submit the assignments at their own pace before the deadlines and were not required to answer all comprehension questions to submit an assignment. Submissions were evaluated by the corresponding teacher using the system. In the first three weeks, students worked on texts at the A2 level, and in weeks four to six on texts at the B1 level. Descriptive statistics of assignments per student (number of assignments assigned, proportion of assignment completed, and time spent per assignment) are shown in Table 3. Before and after the learning period, a proficiency test measuring learners’ L2 English reading proficiency was administered in the system. A questionnaire measuring learners’ motivation toward L2 reading was administered before the learning period. Descriptive statistics of the tests and questionnaire are shown in Table 4.

**Table 3:** Descriptive statistics of assignments per student.

	M	SD
No. of assignments assigned	10.57	1.64
Proportion of assignments completed ( %)	79.28	0.24
Time-on-assignment (min.)*	4116.93	8801.32

*Note. We detected outliers in the time-on-assignment measure. Close examination revealed that these instances were likely caused by students’ unintended behavior, such as system errors and off-task behavior. We treated these outliers with winsorization [8] with a trim quantile of 0.10 (i.e., observations below and above than the fifth and 95th percentiles are replaced by values at the fifth and 95th percentiles respectively).

**Table 4:** Descriptive statistics of the pre-/post-tests and motivation questionnaire.

	N	M	SD	Min	Max	Cronbach’s α
Tests						
Pre-test	186	4.93	2.53	0.00	11.00	0.65
Post-test	149	5.41	3.14	0.00	11.00	0.79
Gain score	142	0.70	3.02	−7.00	9.00	-
Questionnaire						
Intrinsic goal orientation	161	18.89	5.71	4.00	28.00	0.92
Extrinsic goal orientation	161	15.01	4.08	3.00	21.00	0.86
Task value	161	29.22	8.35	6.00	42.00	0.95
Control of learning beliefs	161	19.40	5.19	4.00	28.00	0.88
Self-efficacy	161	22.51	7.25	5.00	35.00	0.95
Test anxiety	161	18.84	5.72	4.00	28.00	0.90

*Note*. Both pre- and post-test scores ranged between 0 and 11 points. The gain score was calculated as the difference between the pre-test score and post-test score. Each item of the questionnaire was rated on a 7-point Likert scale ranging from one (completely disagree) to seven (completely agree).

### 4.2 ICALL system “ARES”

**Fig. 3:**
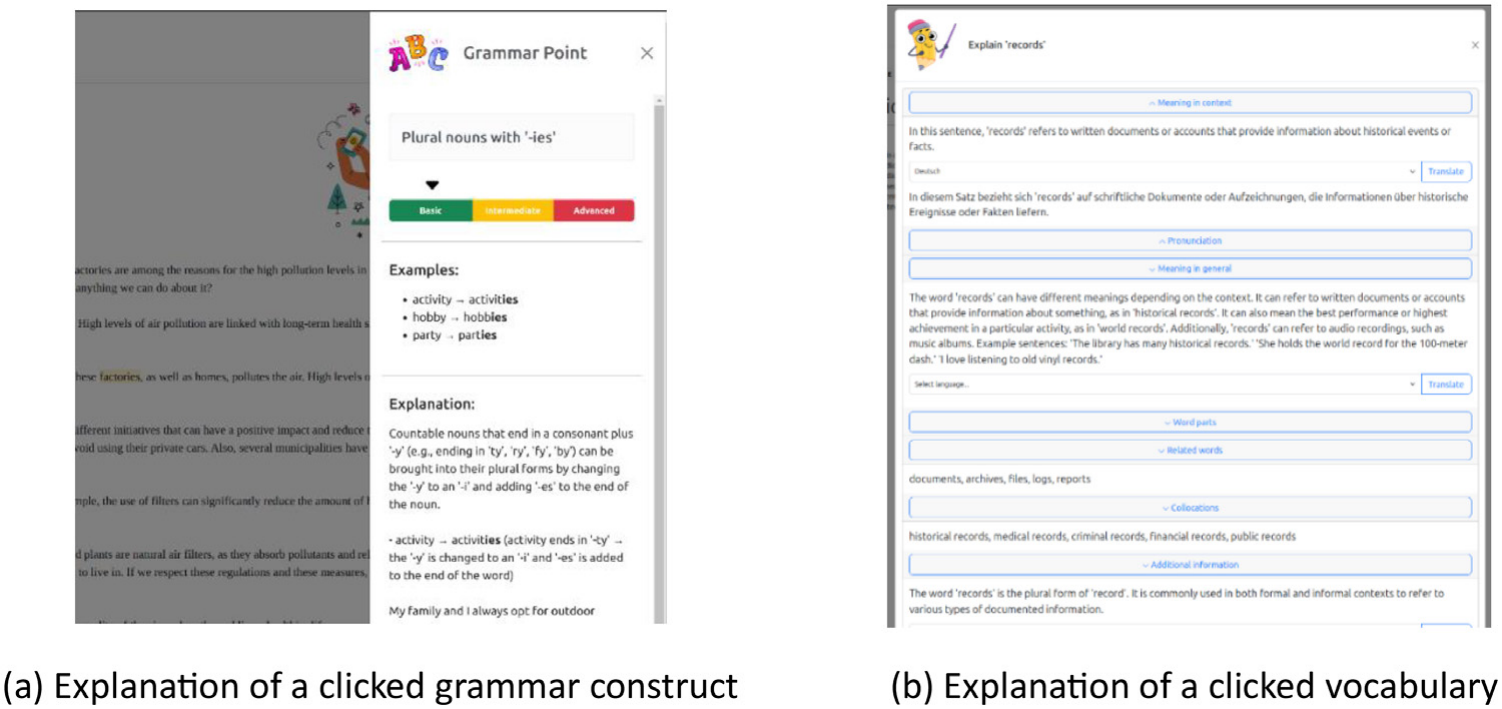
Explanations of a clicked grammar construct and vocabulary.

The study was conducted using *ARES*, a web-based ICALL system to be used by teachers and students for L2 English reading exercises. *ARES* is a pedagogically grounded ICALL system aiming at enhancing L2 reading abilities to be used by teachers and students in formal education settings. By leveraging current language technologies including an in-house Natural Language Processing (NLP) tool and the generative AI tool (ChatGPT^2^), the system equips teachers with a tool that facilitates the easy creation and evaluation of reading activities while offering students interactive and individualized learning support. Specifically, for students, it offers automatic grammar and vocabulary explanations and usage examples that can be consulted as needed by clicking the word during the reading process. As shown in Figs. 3, the system provides students with automatically generated in-context vocabulary and grammar support through annotation panels during text reading. For teachers, the system partially automates the creation of reading comprehension questions and feedback for each student’s responses using the generative AI. This significantly reduces the workload associated with manual grading and feedback generation, allowing them to focus on more communicative activities during the class.

All user interactions with the system such as button clicks, vocabulary lookups, reading comprehension question attempts, and assignment submissions are recorded as interaction logs using xAPI and stored in a Learning Record Store (LRS). More details of the design principles and architectural setup of the system can be found in Lee et al. [7] and Lee [9].

### 4.3 Materials

Students’ L2 English reading proficiency was assessed before and after the learning period using a passage selected from a TOEFL-iBT sample test^3^ on the system, comprising 12 multiple-choice items, each with a single correct answer. A full list of test items is provided in the Supplementary material. Following item and scale analysis using Item Response Theory (IRT) [10], one item was excluded from due to poor psychometric properties, resulting in a final set of 11 items. We computed a sum score for each student (one point per correctly completed item) for each test. Both pre- and post-test scores ranged from 0 to 11 points with acceptable to good internal consistency (see Table 4 for Cronbach’s α of each test). A gain score was calculated as the difference between the pre-test score and post-test score.

A questionnaire assessing students’ motivation toward the subject was administered before the learning period on the system. The items were adapted from the short version of the Motivated Strategies for Learning Questionnaire (MSLQ) by Wang et al. [11], which was originally based on the widely used MSLQ by Pintrich et al. [12] and subsequently adapted and validated to ensure cultural and contextual appropriateness for Chinese students. A full list of questionnaire items is provided in the Supplementary material. The questionnaire comprised subscales, namely intrinsic goal orientation, extrinsic goal orientation, task value, control of learning beliefs, self-efficacy, and test anxiety. Each item was rated on a 7-point Likert scale ranging from one (completely disagree) to seven (completely agree). The subscales of the questionnaire showed good internal consistency (see Table 4 for Cronbach’s α of each subscale of the questionnaire).

### 4.4 Data pre-processing

**Fig. 4:**
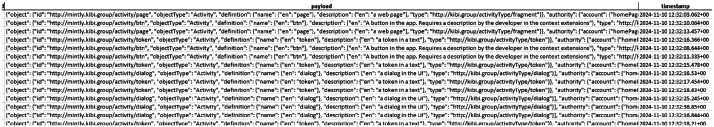
An example of raw pseudonymized interaction logs.

**Fig. 5:**
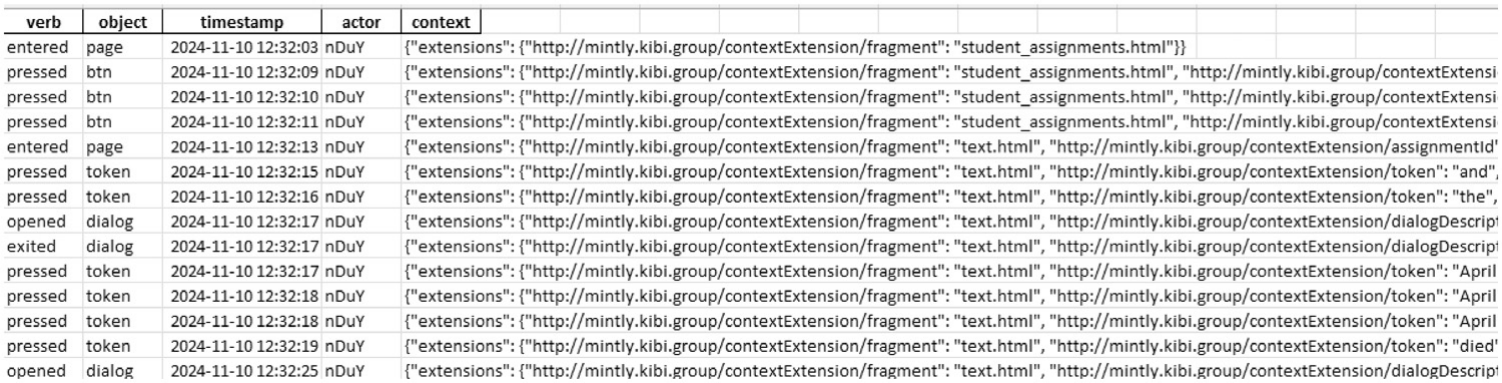
An example of pre-processed pseudonymized interaction logs.

Students’ behavioural data were initially collected as raw unformatted xAPI statements in a JSON format from the LRS (see Fig. 4; the pseudonymized raw xAPI statements are stored in the *payload* column). These raw statements required transformation into an analysable format. In addition, the dataset contained records from users who were not part of the target sample (e.g., test users, inactive users). To ensure data quality, we implemented a three-step preprocessing procedure in Python. First, we applied pseudonymization by replacing identifiable information with pseudonymized codes to protect student privacy. Second, the raw JSON-formatted xAPI statements were transformed into a structured tabular format by extracting key components, such as actor, verb, object, context, and timestamp, and placing them into separate columns for ease of query and analysis. An example of raw interaction logs is provided in Fig. 4, alongside its corresponding cleaned tabular entries in Fig. 5. Finally, we removed data from users who (1) did not provide consent for scientific use, (2) had no active log records in the system, (3) had log records but no assignment submissions, or (4) were included only for testing purposes by the researchers. In the end, the cleaned preprocessed file contained only xAPI statements converted into a structured tabular format and records from consented, active students who had submitted assignments, ensuring the dataset is both analysable and representative of the target sample. For transparency and reproductivity, the raw pseudonymized data, preprocessed data, and scripts used for the data preprocessing are provided in the data repository.

## Potential and Outlook

The dataset presented in this article includes detailed interaction logs, L2 English reading proficiency scores, and self-reported motivation measures. This combination of behavioral and person-level data enables researchers to triangulate multimodel data to examine how factors such as motivation, engagement, and reading proficiency jointly influence learning processes and outcomes. For example, researchers could investigate whether highly motivated learners exhibit more efficient navigation strategies, whether students with higher initial proficiency spend less time on less complex tasks, or how fluctuations in engagement across sessions relate to initial motivation levels in and subsequent learning gains. Furthermore, educators, instructional designers, and learning-system developers may leverage these insights to create adaptive learning interventions and personalized feedback mechanisms. For instance, designers could tailor support based on a learner’s motivation level and engagement patterns across sessions or trigger adaptive scaffolding when behavioral indicators suggest disengagement or difficulty with specific tasks.

## Limitations

While this dataset offers rich multimodal information on learners’ online reading engagement, several limitations should be noted. First, the sample is limited to 201 English majors from a single Chinese university, which may restrict the generalizability of findings. Future research could address this limitation by collecting comparable datasets from learners in other disciplines, institutions, and cultural contexts, thereby enhancing external validity. Second, although the dataset contains detailed clickstream logs, test scores, and self-reported motivation, other relevant constructs such as affective states were not captured. Collecting additional learner characteristics and affective measures in future work would provide a more comprehensive view of learning processes. Finally, the dataset excludes certain contextual details (e.g., teacher practices), which may constrain analyses of social or instructional influences. Future datasets could incorporate such contextual variables to enable richer multilevel analyses.

## Ethics Statement

Prior to the data collection, an ethics approval was granted by an ethics committee of University of Tübingen. The participants provided informed consent for the study participation and use of data for research purposes (see the Supplementary material for the original and English translation of the informed consent). Participants who did not consent were not included in the dataset.

## CRediT Author Statement

**Mihwa Lee:** Writing – original draft, Project administration, Formal analysis, Data curation, Conceptualization, Methodology, Software, Funding acquisition. **Björn Rudzewitz:** Writing – review & editing, Project administration, Conceptualization, Methodology, Software, Funding acquisition. **Yushan Ye, Lanhua Huang, Yuan Chu, and Xiaoer Zhou:** Project administration, Methodology. **Xiaobin Chen:** Writing – review & editing, Project administration, Conceptualization, Methodology, Software, Funding acquisition, Supervision.

## Data Availability

OSFData of interaction logs, learning achievement, and motivation of L2 learners in a computer-assisted language learning environment (Original data).

## Appendix Supplementary materials

Image, application 1

## Footnotes

1 https://xapi.com/.

2 https://chatgpt.com/.

3 https://www.ets.org/pdfs/toefl/toefl-ibt-free-practice-test.pdf.
