# Supplementary material for: Triangulating multimodal data: Data of interaction logs, learning achievement, and motivation of L2 learners in a computer-assisted language learning environment
Source: Data Brief. 2025 Dec 24;64:112426. doi: 10.1016/j.dib.2025.112426 (PMC12813454; doi:10.1016/j.dib.2025.112426)
Supplement: Supplementary file 1 [file mmc1.docx]

**Supplementary material**

**Informed consent (English translation)**

**Invitation to Participate in Research**
Thank you for using our English learning system! We would like to obtain your consent to use your learning data for research purposes. Your data will be used only for research and will never affect your grades. Below is a detailed explanation of the types of data we collect in the study, how they are processed, and the protection measures in place.

**What data will be collected during the study?**
During the research, the system will collect the following data:

- Questionnaire data about your attitudes toward English learning and your learning style
- Pre-test and post-test results
- System usage logs and performance data
- Personal information (e.g., age, gender, educational background, language background)

**How will the collected data be processed?**
All information collected in the study will be kept strictly confidential. Your identity will be anonymized, and the published research results will not contain any information that could directly identify you. The data will be securely protected, and only authorized researchers will have access. Staff who require access to analyze the data must sign a written agreement and comply with data protection regulations. Data will not be shared with third parties without your additional consent.

**Your rights**
Participation in this study is entirely voluntary. Not participating will not result in any negative consequences, especially not in relation to your grade assessment. If you wish to withdraw from the study, you can contact us via the following email addresses: [team@kibi.group](mailto:team@kibi.group) or [mihwa.lee@uni-tuebingen.de](mailto:mihwa.lee@uni-tuebingen.de). Before anonymization (January 12, 2025), you have the right to request access to, correction of, deletion of, restriction of processing of, or to object to the continued processing of your personal data. After anonymization, if you have voluntarily created and provided a personal code word, you may still request deletion of your personal data via email.

**Publication of research results**
The research results will be published in academic journals or conferences in anonymized form, ensuring that under no circumstances can specific individuals be identified from the results.

**Subsequent scientific use**
After the study is completed, anonymized personal data will be transferred—only with your explicit consent—to a professional research data center, in accordance with the guidelines of the German Research Foundation (DFG) on good scientific practice. The data center will ensure secure storage and access protection. These data will be made available to other researchers for scientific purposes in related fields, while maintaining confidentiality and complying with data protection laws.

**Contact information**
If you have any questions about the study, please contact us:

Mihwa Lee, M.A.

Hector-Institut für Empirische Bildungsforschung

Universität Tübingen

[mihwa.lee@uni-tuebingen.de](mailto:mihwa.lee@uni-tuebingen.de)

Prof. Dr. Xiaobin Chen

Hector-Institut für Empirische Bildungsforschung

Universität Tübingen

[xiaobin.chen@uni-tuebingen.de](mailto:xiaobin.chen@uni-tuebingen.de)

If you agree to the conditions of the study, please check the statement below and click the “Submit” button.

“I agree to the conditions of the study.”
[Submit]

**Informed consent (original Chinese version)**

**邀请参与研究**

感谢您使用我们的英语学习系统！我们希望获得您的同意，使用您的学习数据进行研究。您的数据仅用于研究目的，并且绝不会影响您的成绩。以下是我们在研究中收集的数据种类、处理方式以及保护措施的详细说明。

**研究期间会收集哪些数据？**

在研究过程中，系统将收集以下数据：

- 关于您对英语学习态度和学习风格的问卷数据
- 前测和后测成绩
- 系统使用的日志和表现数据
- 个人信息（例如：年龄、性别、教育背景、语言背景）

**收集的数据会如何处理？**

所有研究中收集的信息将被严格保密。您的身份将被匿名化，发布的研究结果不会包含任何可以直接识别您的信息。数据将受到安全保护，仅授权的研究人员可以访问这些数据。需要访问数据进行分析的工作人员必须签署书面协议，遵守数据保护法规。未经您的额外同意，数据不会共享给第三方。

**您的权利**

参与本研究完全是自愿的。不参与研究不会带来任何不利影响，尤其是不会影响您的成绩评估。如需退出研究，可以通过以下电子邮件联系我们：[team@kibi.group](mailto:team@kibi.group) , [mihwa.lee@uni-tuebingen.de](mailto:mihwa.lee@uni-tuebingen.de)。在匿名化完成之前（2025年1月12日），您有权请求访问、更正、删除、限制处理或反对继续处理您的个人数据。在匿名化之后，如果您自愿生成并提供了个人代码词，您仍可以通过电子邮件请求删除您的个人数据。

**研究成果的发布**

研究成果将在学术期刊或会议中以匿名化形式发布，确保任何情况下都无法从研究结果中推断出具体提供信息的人员。

**科学用途的后续使用**

在研究完成后，匿名化的个人数据将在您明确同意的情况下，按照德国研究基金会（DFG）关于良好科学实践的指导方针，转交至专业研究数据中心。数据中心将确保数据的安全存储和访问保护。这些数据将提供给其他研究人员用于相关研究领域的科学目的，同时确保数据的保密性并遵守数据保护法律。

**联系方式**

如果您对研究有任何疑问，请联系我们：

Mihwa Lee, M.A.

Hector-Institut für Empirische Bildungsforschung

Universität Tübingen

[mihwa.lee@uni-tuebingen.de](mailto:mihwa.lee@uni-tuebingen.de)

Prof. Dr. Xiaobin Chen

Hector-Institut für Empirische Bildungsforschung

Universität Tübingen

[xiaobin.chen@uni-tuebingen.de](mailto:xiaobin.chen@uni-tuebingen.de)

如果您同意研究的相关条件，请勾选以下声明并点击“提交”按钮。

“我同意研究条件。”

[提交]

**L2 English reading proficiency test**

Instruction:

- You will read and answer questions about one passage.
- You will have 18 minutes to read the passage and respond to the questions. A clock will indicate how much time is remaining.
- Your answers will be automatically recorded when the time is up. You won't be able to change any answers after that.
- The result does NOT influence your grade, so don't worry!
- You can take this test only ONCE! You can't go back once you start the test.
- If you're ready, press "Start".

Ancient Rome and Greece

1. There is a quality of cohesiveness about the Roman world that applied neither to Greece nor perhaps to any other civilization, ancient or modern. Like the stones of a Roman wall, which were held together both by the regularity of the design and by that peculiarly powerful Roman cement, so the various parts of the Roman realm were bonded into a massive, monolithic entity by physical, organizational, and psychological controls. The physical bonds included the network of military garrisons, which were stationed in every province, and the network of stone-built roads that linked the provinces with Rome. The organizational bonds were based on the common principles of law and administration and on the universal army of officials who enforced common standards of conduct. The psychological controls were built on fear and punishment—on the absolute certainty that anyone or anything that threatened the authority of Rome would be utterly destroyed.

2. The source of the Roman obsession with unity and cohesion may well have lain in the

pattern of Rome’s early development. Whereas Greece had grown from scores of scattered cities, Rome grew from one single organism. While the Greek world had expanded along the Mediterranean Sea lanes, the Roman world was assembled by territorial conquest. Of course, the contrast is not quite so stark: in Alexander the Great the Greeks had found the greatest territorial conqueror of all time; and the Romans, once they moved outside Italy, did not fail to learn the lessons of sea power. Yet the essential difference is undeniable. The key to the Greek world lay in its high-powered ships; the key to Roman power lay in its marching legions. The Greeks were wedded to the sea; the Romans, to the land. The Greek was a sailor at heart; the Roman, a landsman.

3. Certainly, in trying to explain the Roman phenomenon, one would have to place great emphasis on this almost animal instinct for the territorial imperative. Roman priorities lay in the organization, exploitation, and defense of their territory. In all probability it was the fertile plain of Latium, where the Latins who founded Rome originated, that created the habits and skills of landed settlement, landed property, landed economy, landed administration, and a land-based society. From this arose the Roman genius for military organization and orderly government. In turn, a deep attachment to the land, and to the stability which rural life engenders, fostered the Roman virtues: gravitas, a sense of responsibility, peitas, a sense of devotion to family and country, and iustitia, a sense of the natural order.

4. Modern attitudes to Roman civilization range from the infinitely impressed to the thoroughly disgusted. As always, there are the power worshippers, especially among historians, who are predisposed to admire whatever is strong, who feel more attracted to the might of Rome than to the subtlety of Greece. At the same time, there is a solid body of opinion that dislikes Rome. For many, Rome is at best the imitator and the continuator of Greece on a larger scale. Greek civilization had quality; Rome, mere quantity. Greece was original; Rome, derivative. Greece had style; Rome had money. Greece was the inventor; Rome, the research and development division. Such indeed was the opinion of some of the more intellectual Romans. “Had the Greeks held novelty in such disdain as we,” asked Horace in his Epistles, “what work of ancient date would now exist?”

5. Rome’s debt to Greece was enormous. The Romans adopted Greek religion and moral philosophy. In literature, Greek writers were consciously used as models by their Latin successors. It was absolutely accepted that an educated Roman should be fluent in Greek. In speculative philosophy and the sciences, the Romans made virtually no advance on early achievements.

6. Yet it would be wrong to suggest that Rome was somehow a junior partner in Greco-Roman civilization. The Roman genius was projected into new spheres— especially into those of law, military organization, administration, and engineering. Moreover, the tensions that arose within the Roman state produced literary and artistic sensibilities of the highest order. It was no accident that many leading Roman soldiers and statesmen were writers of high caliber.

Directions: Now answer the questions.

1. According to paragraph 1, all of the following are controls that held together the Roman world EXCEPT

A. administrative and legal systems

B. the presence of the military

C. a common language

D. transportation networks

2. According to paragraph 2, which of the following was NOT characteristic of Rome’s early

development?

A. Expansion by sea invasion

B. Territorial expansion

C. Expansion from one original settlement

D. Expansion through invading armies

3. Why does the author mention “Alexander the Great” in the passage?

A. To acknowledge that Greek civilization also expanded by land conquest

B. To compare Greek leaders to Roman leaders

C. To give an example of a Greek leader whom Romans studied

D. To indicate the superior organization of the Greek military

4. The word “fostered” in the passage is closest in meaning to

A. accepted

B. combined

C. introduced

D. encouraged

5. Paragraph 3 suggests which of the following about the people of Latium?

A. Their economy was based on trade relations with other settlements.

B. They held different values than the people of Rome.

C. Agriculture played a significant role in their society.

D. They possessed unusual knowledge of animal instincts.

6. Paragraph 4 indicates that some historians admire Roman civilization because of

A. the diversity of cultures within Roman society

B. its strength

C. its innovative nature

D. the large body of literature that it developed

7. According to paragraph 4, intellectual Romans such as Horace held which of the following opinions about their civilization?

A. Ancient works of Greece held little value in the Roman world.

B. The Greek civilization had been surpassed by the Romans.

C. Roman civilization produced little that was original or memorable.

D. Romans valued certain types of innovations that had been ignored by ancient Greeks.

8. Which of the following statements about leading Roman soldiers and statesmen is supported by paragraphs 5 and 6?

A. They could read and write the Greek language.

B. They frequently wrote poetry and plays.

C. They focused their writing on military matters.

D. They wrote according to the philosophical laws of the Greeks.

9. In the paragraph below, there is a missing sentence. Look at the paragraph and indicate (A, B, C and D) where the following sentence could be added to the passage.

They esteem symbols of Roman power, such as the massive Colosseum.

Where would the sentence best fit?

Modern attitudes to Roman civilization range from the infinitely impressed to the thoroughly disgusted. (A) As always, there are the power worshippers, especially among historians, who are predisposed to admire whatever is strong, who feel more attracted to the might of Rome than to the subtlety of Greece. (B) At the same time, there is a solid body of opinion that dislikes Rome. (C) For many, Rome is at best the imitator and the continuator of Greece on a larger scale. (D) Greek civilization had quality; Rome, mere quantity. Greece was original; Rome, derivative. Greece had style; Rome had money. Greece was the inventor; Rome, the research and development division. Such indeed was the opinion of some of the more

intellectual Romans. “Had the Greeks held novelty in such disdain as we,” asked Horace in his Epistles, “what work of ancient date would now exist?”

A. Option A

B. Option B

C. Option C

D. Option D

10. Directions: An introductory sentence for a brief summary of the passage is provided below. Complete the summary by selecting the THREE answer choices that express the most important ideas in the passage. Some sentences do not belong in the summary because they express ideas that are not presented in the passage or are minor ideas in the passage. This question is worth 2 points.

Write your answer choices in the spaces where they belong. You can either write the letter of your answer choice or you can copy the sentence.

The Roman world drew its strength from several important sources.

Answer Choices

A. Numerous controls imposed by Roman rulers held its territory together.

B. The Roman military was organized differently from older military organizations.

C. Romans valued sea power as did the Latins, the original inhabitants of Rome.

D. Roman values were rooted in a strong attachment to the land and the stability of rural life.

E. Rome combined aspects of ancient Greek civilization with its own contributions in new areas.

F. Educated Romans modeled their own literature and philosophy on the ancient Greeks.

Snapshot of the test:


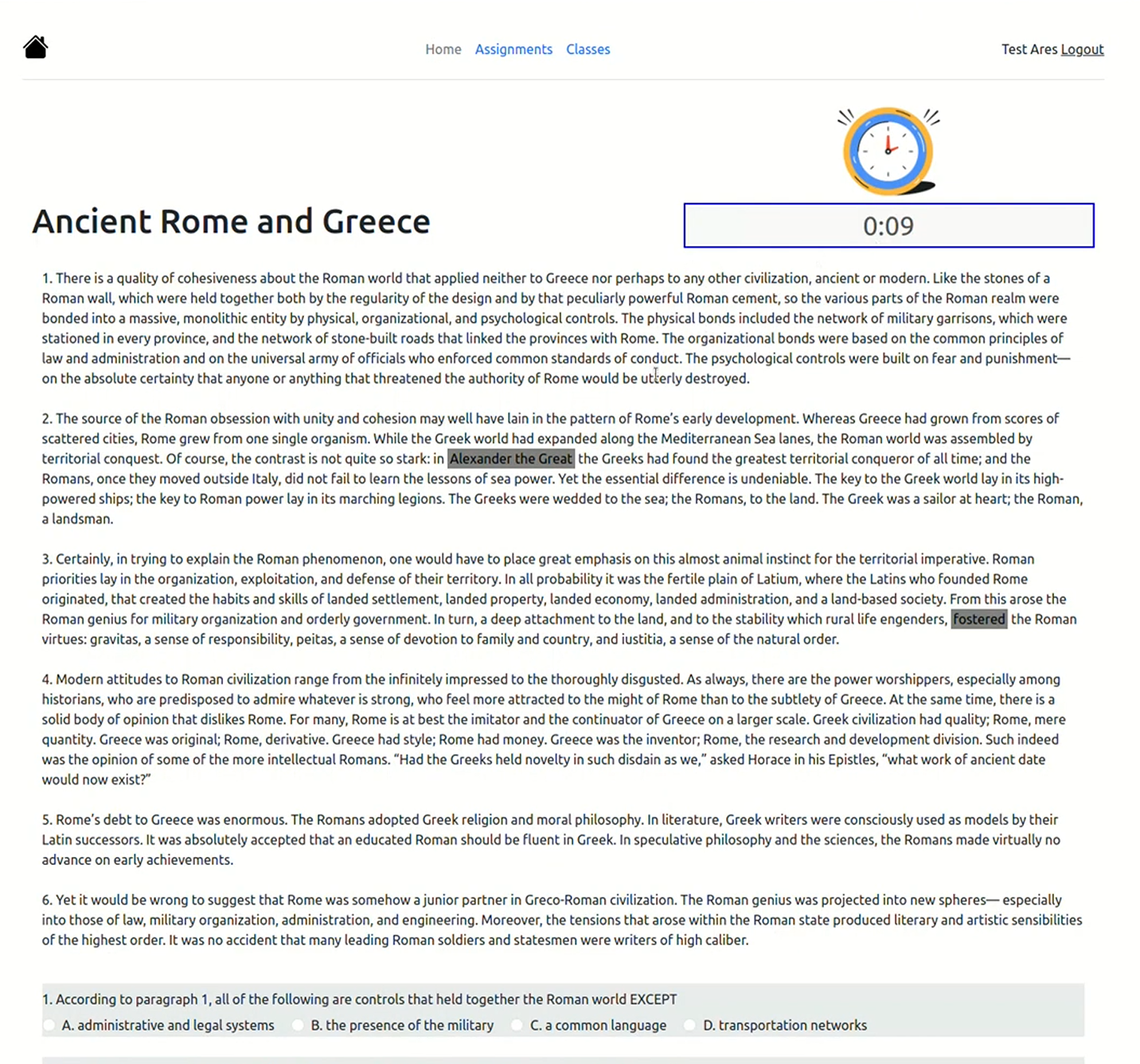


**Motivation questionnaire**

Instruction:

- In this part, we would like you to tell us how much you agree or disagree with the following statements by simply choosing a number from 1 (strongly disagree) to 7 (strongly agree). Please do not leave out any item.

(请根据以下的问题选择相应的数字代表同意或不同意的程度。1表示强烈不赞同，7表示强烈赞同。)

*Intrinsic goal orientation*

1. In the English reading class, I prefer course material that really challenges me so I can learn new thing.

在英语阅读课上，我喜欢那些具有挑战性的学习内容，这样我可以学到新的东西。

2. In the English reading class, I prefer course material that arouses my curiosity, even if it is difficult to learn.

在英语阅读课上，我更喜欢学习那些能够引起我学习兴趣的内容，即使它比较难学。

3. The most satisfying thing for me in the English reading course is trying to understand the content as thoroughly as possible.

英语阅读课最让我满意的地方是我能够尽可能透彻地理解它的内容。

4. When I have the opportunity in the English reading class, I choose course assignments that I can learn from even if they don’t guarantee a good grade.

在英语阅读课上，如果有选择的话，我会选择那些能让我学到更多东西的作业，即使这些作业不能保证我得到高分。

*Extrinsic goal orientation*

5. Getting a good grade in the English reading class is the most satisfying thing for me right now.

在英语阅读课上拿到好分数是目前最令我满意的事。

6. The most important thing for me right now is improving my overall grade point average, so my main concern in the English reading class is getting a good grade.

现在对我来说最重要的事情就是成绩，所以我对英语阅读课的主要期望是要拿个好成绩。

7. If I can, I want to get better grades in the English reading class than most of the other students.

如果可能的话，我希望我在英语阅读课拿到比班里大部分同学更高的分数。

*Task value*

8. I think I will be able to use what I learn in the English reading course in other courses.

我认为我能够把在英语阅读课上的所学应用到其它的学科之中。

9. It is important for me to learn the course material in the English reading class.

对我来说，学好英语阅读课上老师讲授的内容非常重要。

10. I am very interested in the content area of the English reading course.

我对英语阅读课上所学的内容很感兴趣。

11. I think the course material in the English reading class is useful for me to learn.

英语阅读课上所学的东西对我来说是有用的。

12. I like the subject matter of the English reading course.

我喜欢英语阅读课所学习的内容。

13. Understanding the subject matter of the English reading course is very important to me.

理解英语阅读课的学习内容对我来说很重要。

*Control of learning beliefs*

14. If I study in appropriate ways, then I will be able to learn the course material in the English reading course.

如果我学习得法，我就能够学会英语阅读课的内容。

15. It is my own fault if I don’t learn the material in the English reading course.

没有学好英语阅读课是我自身的问题。

16. If I try hard enough, then I will understand the English reading course material.

如果我足够努力，我就能够理解英语阅读课学习材料。

17. If I don’t understand the English reading course material, it is because I didn’t try hard enough.

如果我不能理解英语阅读课程学习材料，那是因为我不够努力。

*Self-efficacy for learning and performance*

18. I believe I will receive an excellent grade in the English reading class.

我相信我在英语阅读课上能够获得一个漂亮的分数。

19. I’m confident I can understand the most complex material presented by the instructor in the English reading course.

我确信自己能够理解英语阅读课上老师所呈现的最复杂的材料。

20. I’m confident I can do an excellent job on the assignments and tests in the English reading course.

我敢肯定我能够出色地完成英语阅读课老师布置的作业和测试。

21.I’m certain I can master the skills being taught in the English reading class.

我确信自己能够掌握英语阅读课上所教授的技能。

22. Considering the difficulty of the English reading course, the teacher, and my skills, I think I will do well in the English reading class.

综合考虑英语阅读课的难度，老师和我自己的能力，我想我可以在英语阅读课上表现得很好。

*Test anxiety*

*23*. When I take an English reading test, I think about items on other parts of the test I can’t answer.

英语阅读课考试时，我一直想着自己不会回答的题目。

24. When I take English reading tests I think if the consequences of failing.

英语阅读课考试时，我会去想挂科的后果。

25. I have an uneasy, upset feeling when I take an English reading exam.

英语阅读课考试时，我会感到紧张不安。

26. I feel my heart beating fast when I take an English reading exam.

英语阅读课考试时，我会感到自己心跳加速。
